# SAG-QC: quality control of single amplified genome information by subtracting non-target sequences based on sequence compositions

**DOI:** 10.1186/s12859-017-1572-5

**Published:** 2017-03-04

**Authors:** Toru Maruyama, Tetsushi Mori, Keisuke Yamagishi, Haruko Takeyama

**Affiliations:** 1 Department of Life Science & Medical Bioscience, Graduate School of Advanced Science & Engineering, Waseda University, 3-4-1 Okubo, Shinjuku, Tokyo, 169-8555 Japan; 2 Computational Bio-Big Data Open Innovation Lab., National Institute of Advanced Science and Technology, 3-4-1 Okubo, Shinjuku, Tokyo, 169-0072 Japan; 3 Institute for Nanoscience and Nanotechnology, Waseda University, 513 Waseda-Tsurumaki-cho, Shinjuku, Tokyo, 162-0041 Japan

**Keywords:** Single-cell genomics, Decontamination, GUI software

## Abstract

**Background:**

Whole genome amplification techniques have enabled the analysis of unexplored genomic information by sequencing of single-amplified genomes (SAGs). Whole genome amplification of single bacteria is currently challenging because contamination often occurs in experimental processes. Thus, to increase the confidence in the analyses of sequenced SAGs, bioinformatics approaches that identify and exclude non-target sequences from SAGs are required. Since currently reported approaches utilize sequence information in public databases, they have limitations when new strains are the targets of interest. Here, we developed a software SAG-QC that identify and exclude non-target sequences independent of database.

**Results:**

In our method, “no template control” sequences acquired during WGA were used. We calculated the probability that a sequence was derived from contaminants by comparing k-mer compositions with the no template control sequences. Based on the results of tests using simulated SAG datasets, the accuracy of our method for predicting non-target sequences was higher than that of currently reported techniques. Subsequently, we applied our tool to actual SAG datasets and evaluated the accuracy of the predictions.

**Conclusions:**

Our method works independently of public sequence information for distinguishing SAGs from non-target sequences. This method will be effective when employed against SAG sequences of unexplored strains and we anticipate that it will contribute to the correct interpretation of SAGs.

**Electronic supplementary material:**

The online version of this article (doi:10.1186/s12859-017-1572-5) contains supplementary material, which is available to authorized users.

## Background

Accessing genetic information in environmental bacteria has been considered challenging, as >99% of currently known microbes cannot be cultivated using current standard cultivation techniques. However, understanding these uncultivable bacteria has been made possible by the whole genome amplification (WGA) techniques that enable the amplification of DNA from as low as several femto-grams. Thus far, WGA methods, particularly multiple displacement amplification (MDA), have promoted the sequencing of bacterial genomes at the single-cell level [1, 2] and have assisted in elucidating the characteristics of several uncultivable taxonomic groups [3–6].

Despite its advantages, WGA of single cells is extremely sensitive and is easily affected by DNA contamination from the surrounding environment. Efforts to eradicate or avoid these contaminants include the use of clean rooms or clean-up techniques [3, 7–11], but it remains difficult to completely remove these contaminants. The presence of contaminating DNA may cause the misinterpretation on the characteristics of the target bacterium. Therefore, quality control of whole genome amplified bacterial genomes, known as single-cell amplified genomes (SAGs), to identify and remove sequences derived from contaminating constituents is critical for subsequent SAG analyses.

Currently, two main approaches are used for the quality control of SAGs. These include approaches dependent on (1) similarity searching and (2) sequence composition. The first approach excludes sequences highly similar to sequences that originated from contaminant species [12]. These methods are highly effective when the target or contaminant species belong to taxonomic groups whose genome information is substantially available. However, these methods are not appropriate when the targets belong to minor taxonomic groups that have not been well-studied. The second approach clusters sequences based on sequence compositions such as tetramer frequencies and then extracts clusters corresponding to the target genome sequences [13]. In contrast to the similarity searching-based approach, this approach enables the removal of contaminant sequences independently of existing information. Several composition-based methods have been proposed for binning of metagenomic information [14–16]. However, even if sequences are grouped into clusters based on sequence composition, there is a difficulty and uncertainty to determine whether the clusters correspond to the target bacterium.

ProDeGe is the first system that achieved fully automated quality control of SAG information [17]. This tool utilizes both similarity searching and sequence composition. However, performance of the tool depends on the results of the similarity search. There are still limitations when a target belongs to a minor uncultivable taxonomic group and there is no sequence of closely related species in a reference database.

In this study, we introduce SAG-QC, a software aimed for the quality control of bacterial SAG sequences. Both approaches based on similarity searching and sequence compositions are available in this application. Unlike the methods for binning of metagenome sequences based on sequence compositions [14–16], SAG-QC identifies clusters of target sequences by utilizing “non-target sequences” that can be acquired experimentally. The non-target sequences are acquired from sequence libraries subjected to experimental processes without template DNA. Therefore, this tool is applicable for minor taxonomic groups for which limited information is available because the function is only dependent on the non-target sequences. Additionally, this application provides a user-friendly graphical user interface, supporting users to remove contaminant sequences intuitively and rapidly. We believe this application enables users to examine various types of SAG sequences.

## Implementation

SAG-QC is a user-friendly graphical user interface application developed using Python. All of the executable files and the source codes are available in https://sourceforge.net/projects/sag-qc. This application is available for Mac OS X.

### Overview of quality control with SAG-QC

**Fig. 1:**
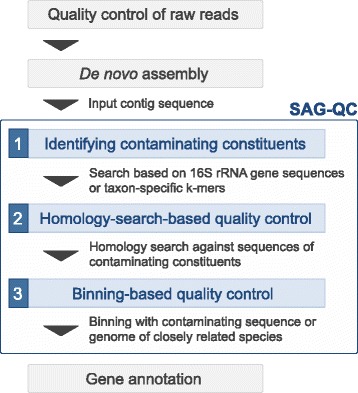
Overview of SAG-QC SAG-QC. Uses contig sequences generated by *de novo* assembly. Decontamination of the contig sequences involves three steps: identification of contaminating constituent, annotation-based decontamination, and binning-based decontamination. The output can be reliably utilized for subsequent downstream analyses such as gene annotation

SAG-QC is designed to exclude contaminant sequences from contigs. SAG-QC includes three steps for the quality control of the input contig sequences (Fig. 1). The first step is the identification of contaminant constituents based on 16S rRNA gene sequences or with a k-mer-based taxonomic classification tool [18]. In the second step, contaminant sequences can be removed by similarity searching against the genome sequence if the genome sequences of the contaminant constituents were determined previously. The third step is a quality control that includes binning based on sequence composition. This step removes contaminant sequences that were not identified in the second quality control step. In this step, contaminant sequences are removed by comparison of the sequence composition to non-target sequences. Non-target sequences can be acquired by sequencing of samples subjected to experimental processes (ex: WGA) without using cells as a negative control. Therefore, this step removes contaminant sequences even when target or contaminant sequences belong to taxonomic groups with limited available genetic information.

### Description of the use of SAG-QC

**Fig. 2:**
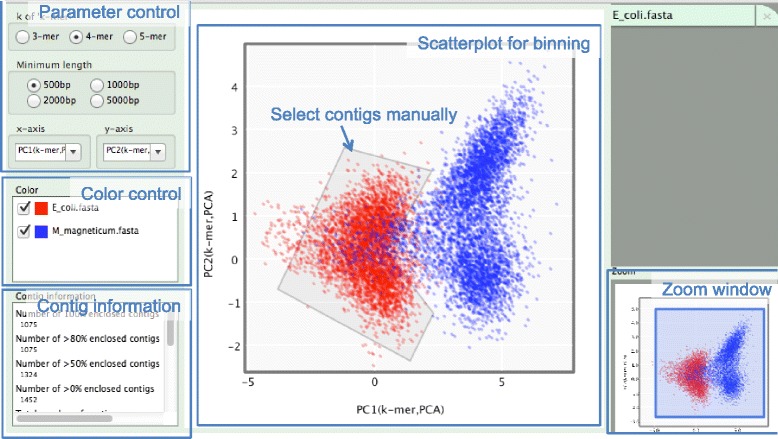
Graphical user interface of SAG-QC. Contigs are plotted as *dots* in the scatterplot based on sequence properties such as GC contents and k-mer frequencies. Users can enclose any regions manually and extract/remove contigs inside the region. Statistics of the enclosed contigs is in the “contig information” panel. Colors of the plots can be edited in the “color control” panel. Users can modify the parameters for drawing the scatterplot in the “parameter control” panel

Input files for SAG-QC are contig sequences in Fasta format. SAG-QC divides the input sequences into small fragments (fragments of 1000 bp in default) and projects them onto a scatterplot based on sequence composition (Fig. 2). The colors of the plots can be changed based on other information (ex: taxonomic information) using the color control panel. Importantly, SAG-QC has a function to extract sequences at any region within the scatterplot by manually clicking and gating the region of interest. This feature allows users to specifically select the region of interest and focus on these regions for downstream analyses.

### Identification of contaminating constituents

**Fig. 3:**
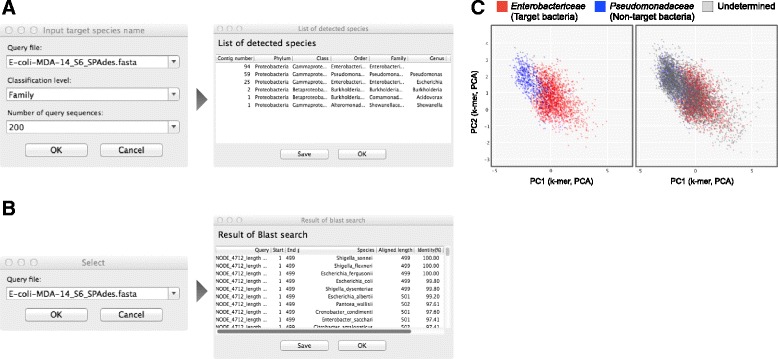
Detection of contaminating constituents. **a** Users can identify contaminating constituents using a k-mer based annotation tool Kraken [16]. **b** Contaminating constituents also can be detected by annotation of 16 rRNA gene sequences. **c** Colors of plots can be set based on classification. Users can extract sequences in a cluster of some specific bacterial species

The subsequent sections include detailed descriptions of the three quality control steps of SAG-QC. In the first step, SAG-QC identifies contaminating constituents using two methods. The first method uses Kraken [18], which is a tool that assigns taxa to sequences based on their characteristic k-mers. Kraken identifies whether the query file includes sequences from contaminating constituents (Fig. 3a). Based on the classification results, users are able to exclude sequences to which unexpected taxa are assigned. The second method is based on the annotation of 16S rRNA gene sequences. SAG-QC utilizes HMMER to predict 16S rRNA gene sequences in the input contigs. The 16S rRNA gene sequences are then annotated by BLAST searching against the SILVA database [19] (Fig. 3b). This step also enables users to identify clusters of target and contaminant sequences by setting the colors of the plots according to the classification results (Fig. 3c).

### Quality control based on similarity search

In the second step, SAG-QC removes contaminant sequences based on similarity searching against the genome sequence of contaminating constituents determined in the previous step. This step is applicable if a SAG sample is contaminated with bacterial DNA whose information is available. SAG-QC uses the similarity search tool BLAT [20] to identify and exclude sequences that show high homology to sequences of contaminating constituents. Any sequence files can be loaded as a database for the similarity search.

### Quality control based on binning with sequence compositions

In the third step, SAG-QC excludes contaminant sequences by comparing sequence compositions with those of non-target sequences. The sequence compositions utilized by SAG-QC are GC contents, principal components of k-mer frequencies, and those of codon frequencies. We used relative synonymous codon usage frequencies as an indicator of codon usage [21]. This implies

$$ {r}_{ij} = \frac{n_j{x}_{ij}}{{\displaystyle {\sum}_{k=1}^{n_j}}{x}_{ik}} $$

where *r*_*ij*_ is relative synonymous codon usage frequency for codon *j* of sequence *i. n*_*j*_ is the number of codons synonymous with codon *j, x*_*ij*_ is the number of codon *j* observed in the sequence *i*. The frequency is calculated for coding sequences predicted using MetaGeneAnnotator [22].

In SAG-QC, multiple sequence files can be projected on a single scatterplot. Thus, clusters of target sequences can be determined by projecting non-target sequences onto the scatterplot together. Additionally, if the genome sequence of species that is closely related to a target bacterium is available, a target cluster can also be identified by loading the genome sequence onto the scatterplot.

**Fig. 4:**
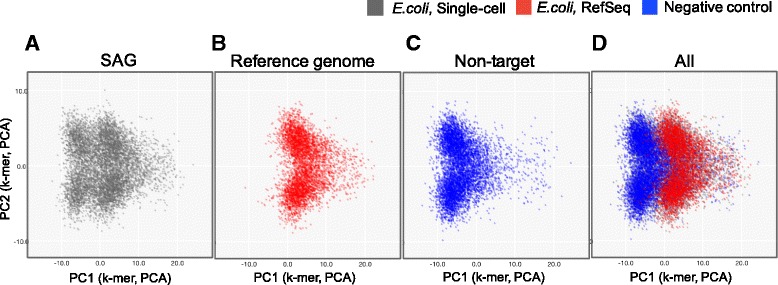
Detection of target clusters using non-target sequence and genome sequence of related species. **a** Scatterplots of contigs generated from single amplified genome (SAG) sequences of *Escherichia coli*. **b** Scatterplot of published genome sequences of *E. coli* in RefSeq. **c** Scatterplot of non-target sequences. **d** Scatterplot in which all sequences are overlapped

For example, we used SAG sequences from *Escherichia coli*. We simultaneously performed WGA without a sorted cell and acquired non-target sequences. These sequences were assembled and projected using the genome sequence of *E. coli* downloaded from RefSeq onto a scatterplot together. Consequently, we found that the distribution of the SAG sequences was composed of two clusters: a cluster of target sequences and a cluster of non-target sequences (Fig. 4). This observation suggests that sequences of either closely related species or non-target sequences can be utilized for identifying the distribution of the target sequences.

### Estimation of confidence scores by utilizing non-target sequences

We established a method utilizing non-target sequences to extract sequences derived from target bacterium with high probability. Similar to the above observation, distributions of SAG sequences can be decomposed into distributions of target and non-target sequences (Fig. 4). SAG-QC possesses a function for predicting where target sequences are distributed on a scatterplot by subtracting the distribution of non-target sequences from that of SAG sequences. Based on the predicted distribution of target sequences, SAG-QC assigns the sequences confidence scores, which are probabilities indicating whether the sequences originated from the target species. These scores help users extract sequences derived from target species with high probability.

*f* ^(*s*)^, *f* ^(*t*)^, and *f* ^(*n*)^denote functions for the probability density of SAG sequences, target sequences, and non-target sequences, respectively. The probability density function of the sample sequences *f* ^(*s*)^ can be decomposed into that of target and non-target sequences. This implies

$$ {f}^{(s)}\left(\boldsymbol{x}\right) = {p}^{(t)}{f}^{(t)}\left(\boldsymbol{x}\right) + {p}^{(n)}{f}^{(n)}\left(\boldsymbol{x}\right) $$

$$ {p}^{(t)} + {p}^{(n)}=1 $$

where ***x*** represents the coordinates in the scatterplots. *p* ^(*t*)^ and *p* ^(*n*)^ are proportions of target and non-target sequences in the sample SAG sequences.

**Fig. 5:**
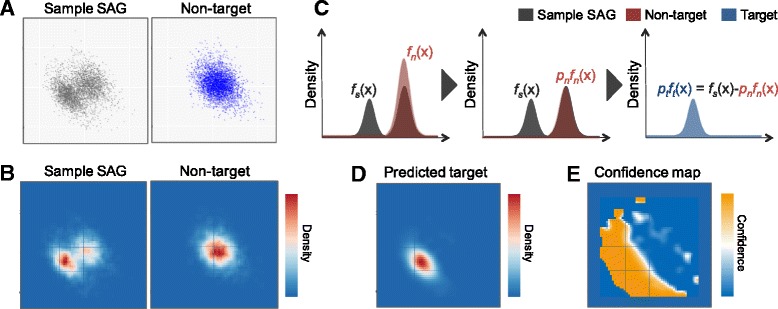
Inference of highly confident region on scatterplot using non-target sequence**. a** Scatterplot based on sequence composition. **b** Heat map showing density of the plots. The densities were calculated by Gaussian kernal distribution estimation. **c** Sample sequences were composed of two constitutions target sequences and contaminating sequences. The distribution of target sequences ideally can be estimated by subtracting the distribution of non-target sequences. **d** Estimated distribution of target sequence. **e** Example of confidence map. The map shows confidence, an indicator reflecting whether a sequence was derived from a target sequence. Users can extract highly confident regions based on this information

*f* ^(*s*)^ and *f* ^(*n*)^ are approximated from the scatterplots. The scatterplot is divided into *M* × *M* blocks (default as *M* = 50). Thereafter, a density matrix *D* is computed based on Gaussian kernel distribution estimation (Fig. 5b). This implies

$$ D{\hbox{'}}_{ij} = \frac{1}{ n h}\ {\displaystyle \sum_{k=1}^n} K\left(\frac{x_{ij}-{x}_k}{h}\right) $$

$$ K(x) = \frac{1}{\sqrt{2\pi}} \exp \left(-\frac{1}{2}{x}^2\right) $$

$$ {D}_{ij} = \frac{D{\hbox{'}}_{ij}}{{\displaystyle {\sum}_{m=1}^M}{\displaystyle {\sum}_{n=1}^M} D{\hbox{'}}_{n m}} $$

*n* corresponds to a number of plots on the scatterplot. *x*_*ij*_ denotes a coordinate at the center of *block*(*i,j*) (block at row *i* and column *j*). *D*_*ij*_ denotes the density of the plots in *block*(*i,j*) and is represented by the probability density at coordinate *x*_*ij*_. The probability density function *f*(***x***) is approximated with the density matrix *D*.

$$ \boldsymbol{x}\in block\left( i, j\right)\Rightarrow f\left(\boldsymbol{x}\right) = {D}_{ij} $$

The bandwidth *h* for kernel density estimation is calculated according to Scott’s rule [23] as follows.

$$ h={n}^{\left(\frac{-1}{d+4}\right)}={n}^{-\frac{1}{6}} $$

SAG samples ideally include fewer contaminant sequences than samples subjected to experimental processes without template DNA (Fig. 5c). This is because the SAG sequences contain target sequences other than the non-target sequences. *p* ^(*n*)^ and *p* ^(*t*)^ are estimated as follows.

$$ \begin{array}{c}\hfill {p}^{(n)}={\displaystyle \sum_{\left( i, j\Big|{D^{(n)}}_{ij}>{D^{(s)}}_{ij}\right)}}{D^{(s)}}_{ij}/{\displaystyle \sum_{\left( i, j\Big|{D^{(n)}}_{ij}>{D^{(s)}}_{ij}\right)}}{D^{(n)}}_{ij}\hfill \\ {}\hfill {p}^{(t)}=1-{p}^{(n)}\hfill \end{array} $$

*D* ^(*s*)^ and *D* ^(*n*)^ correspond to the distribution of SAG sequences and that of non-target sequences, respectively. The distribution of target sequences *D* ^(*t*)^ is estimated by subtracting distribution *D* ^(*n*)^ from that of *D* ^(*s*)^ as follows (Fig. 5d).

$$ {D^{(t)}}_{ij} = \left({D^{(s)}}_{ij} - {p}^{(n)}{D^{(n)}}_{ij}\right)/{p}^{(t)} $$

$$ x\in block\left( i, j\right)\Rightarrow {f}^{(t)}(x)={D^{(t)}}_{ij} $$

Based on these parameters, confidence *c* is calculated as follows. Confidence score *c*(*x*) denotes a probability that a sequence plotted on *x* is a target sequence.

$$ c(x)=\frac{p^{(t)}{f}^{(t)}(x)}{p^{(t)}{f}^{(t)}(x) + {p}^{(n)}{f}^{(n)}(x)} $$

Based on the confidence scores, users can draw a confidence map (Fig. 5e). A confidence map indicates regions that are highly dense with target sequences on a scatterplot. Let *x*_*ij*_ denote the coordinate of the *j*_th_ fragment of sequence *i*, confidence scores of the sequence *c*_*i*_ are calculated as follows. *n*_*i*_ is the number of fragments originated from sequence *i*.

$$ {c}_i=\frac{{\displaystyle {\sum}_{j=1}^{n_i}} c\left({x}_{i j}\right)}{n_i} $$

Confidence scores can be utilized as thresholds for the extraction of sequences. If confidence score of 0.8 is set as a threshold, we can extract sequences originated from target bacterium with a probability of more than 80%. Additionally, the scores can be utilized to support the results of subsequent genome analyses. For example, genes found on sequences with high confidence scores are more reliable than those on sequence with low scores.

## Methods

### Sequencing SAG of *E. coli*

*Escherichia coli* K-12 were cultured overnight at 37 °C in LB broth. *Escherichia coli* cells were washed with nuclease-free water and stained with SYTO9 Green Fluorescent Nucleic Acid Stain (Life Technologies, Carlsbad, CA, USA). Single cells were sorted using FACS Aria II (BD Biosciences, Franklin Lakes, NJ, USA) with a 488-nm laser and forward scatter light. Genome extraction and MDA were conducted using the Genomiphi V2 Amplification Kit (GE Healthcare, Little Chalfont, UK) according to the manufacturer’s protocol. The amplified products were screened by sequencing the 16S rRNA genes. PCR amplifications of 16S rRNA genes were performed using universal primer 27F-338R. The PCR products were sequenced by Sanger sequencing. Taxonomic classifications were conducted by BLAST searching against the NCBI nr database. We have prepared three MDA products whose 16S rRNA genes are annotated as *E. coli* were selected for the following steps. In the meantime, to acquire non-target sequences, we performed MDA without template DNA of *E. coli*.

The MDA products were purified with Zymo Research Genomic DNA Clean & Concentrator-10 (Zymo Research, Irvine, CA, USA). Debranching was conducted on the purified samples with S1 nuclease (TaKaRa, Shiga, Japan). Thereafter, the samples were purified again using the Zymo Research Genomic DNA Clean & Concentrator-10. Sequence libraries were prepared with Nextera XT (Illumina, San Diego, CA, USA). The libraries were sequenced on an Illumina MiSeq in 2 × 300 bp mode. We sequenced three SAGs of *E. coli* and single no-template MDA product in this experiment.

### Preprocessing of SAG sequences

The sequence reads were preprocessed through several steps. We first removed reads whose half of quality scores was below 25 using the fastx-toolkit (fastq_quality_filter –q 25 –p 50). Sequence regions with quality scores below 20 were trimmed from the 3′ end by using PRINSEQ (prinseq-lite.pl –trim_qual_right 20) [24]. Sequence reads including ambiguous bases (“N”) more than 1% of the whole were also discarded using PRINSEQ (prinseq-lite.pl -ns_max_p 1). Sequence reads shorter than half of the average read length were removed using an in-house python script. Finally, we excluded reads whose pair-reads were discarded using the above steps with an in-house R script.

### *De novo* assembly of SAG sequences

We conducted *de novo* assembly of the preprocessed sequence reads using SPAdes [25]. We set options recommended for assembling from sequences amplified through MDA (spades.py --sc --careful --disable-rr). Contig sequences shorter than 500 bp were discarded from subsequent analyses.

### Performance test with simulated SAG sequences

SAG sequences were simulated using publicly available genome sequences. Two bacterial species, *E. coli* and *Magnetospirillum magneticum*, were used as target species in this simulation. These bacteria were selected to examine whether this method is applicable to wide variety of species because their genomes exhibit quite different GC contents. Their genome sequences NC_000913 (*E. coli*) and NC_007626 (*M. magneticum*) were downloaded from the NCBI Genome database (ftp://ftp.ncbi.nlm.nih.gov/genomes/refseq/bacteria/). Contigs of the target bacteria were simulated by sampling sequence fragments randomly from the genome sequences. We also downloaded all genome sequences of genera *Pseudomonas* and *Delftia*, which are commonly observed as contaminants in SAG sequences [7]. Non-target sequences were simulated by randomly sampling sequence fragments from the genome sequences of *Pseudomonas* and *Delftia*. The average and standard deviations of the sampled sequence lengths were set 3000 bp and 500 bp, respectively. Subsequently, the datasets simulated as target and non-target sequences were mixed in several different proportions to simulate contaminated SAG sequences (Table 1). Total numbers of simulated SAG sequences were set to 1000 for all proportions of contamination.

**Table 1:** Number of target and non-target sequences in simulated SAG data

Target	Contamination		Proportion of contaminant sequence [%]
*E. coli/M. magneticum*	*Pseudomonas*	*Delftia*	
1000	0	0	0
900	75	25	10
800	150	50	20
700	225	75	30
600	300	100	40
500	375	125	50
400	450	150	60
300	525	175	70
200	600	200	80
100	675	225	90
0	750	250	100

We utilized public bacterial sequences to simulate SAG datasets. We defined *Escherichia coli* and *Magnetospirillum magneticum* as target species in this simulation. We mixed their sequences with sequences of *Pseudomonas* and *Delftia* to simulate sequences of contaminated samples. The sequences were mixed in several proportions to simulate datasets with different contamination levels.

Using the simulated SAG sequences, we examined the accuracy of estimating the contamination rate *p* ^(*n*)^, distribution of target sequences *f* ^(*t*)^, and confidence scores. The simulated SAG sequences, target sequences (contamination rate 0%), and non-target sequences (contamination rate 100%) were respectively divided into fragments of 1000 bp and projected on a single scatterplot. The proportions of non-target sequences *p* ^(*n*)^ and distributions of target sequences *D* ^(*t*)^ were predicted by subtracting the distribution of non-target sequences *D* ^(*n*)^ from that of sample SAG sequences *D* ^(*s*)^. The accuracy of predicted proportions *p* ^(*n*)^ was evaluated by examining the correlations with simulated contamination rates. The accuracy of the predicted distribution *D* ^(*t*)^ was evaluated from the correlation with the distribution of the simulated target sequences. Thereafter, we calculated confidence scores based on the predicted distributions *D* ^(*t*)^ and distributions of the non-target sequences *D* ^(*n*)^. To assess the potential of the confidence score to distinguish between target sequences and contaminant sequences, receiver operating characteristic (ROC) curves were generated and areas under curves (AUC) were calculated with R package pROC [26]. We constructed simulated datasets and conducted performance tests 5 times.

### Performance test using real SAG sequences

We evaluated the accuracy of predicting the distribution of target sequences *D* ^(*t*)^ by utilizing real SAG sequences that were acquired from *E. coli* experimentally. Non-target sequences were simultaneously collected from sequence libraries that were amplified by MDA without template DNA. Contigs of both *E. coli* SAG sequences and non-target sequences were divided into fragments of 1000 bp and mapped onto a scatterplot together. Additionally, we randomly sampled 1000 sequences from a published genome sequence of *E. coli* and projected them onto the scatterplot. The average length and standard deviation of the sequence lengths were set to 3000 bp and 500 bp, respectively. The distribution of target sequences *D* ^(*t*)^ was predicted based on the sample SAG sequences and non-target sequences. The accuracy of the prediction was evaluated by comparing the predicted distribution *D* ^(*t*)^ and the distribution of the published genome sequence.

To evaluate the performance of the confidence score, we attempted to identify target sequences from the contig sequences. Contigs of the SAG sequences were aligned with the genome sequence of *E. coli* using MUMmer [27]. We regarded contigs as target sequences if the regions that aligned with the *E. coli* genome were longer than the half-length of the contigs. Based on the alignment results, the performance of the confidence score was evaluated. Confidence scores were computed from the predicted distribution *D* ^(*t*)^ and the distribution of non-target sequences *D* ^(*n*)^. We generated ROC curves to examine whether the confidence scores correctly reflected the annotation results.

## Results and discussion

### Performance test using simulated SAG sequences

**Fig. 6:**
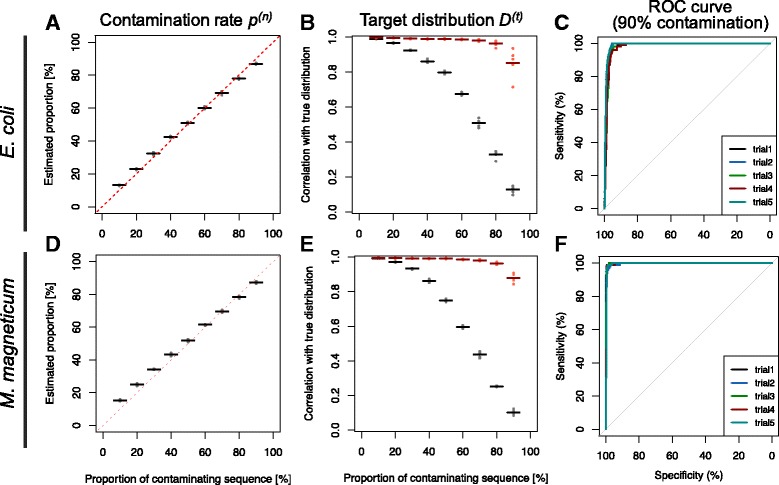
Results of performance test using simulated SAG. **a**, **d** Plot representing performance of estimating contamination rate. **b**, **e** Plot showing capacity for predicting distribution of target sequences. The vertical axis represents Pearson’s correlation with true distribution. **c**, **f** ROC curve showing accuracy for the prediction of a target sequence

The accuracy of our method for predicting the proportion of non-target sequences *p* ^(*n*)^ and distribution of target sequences *D* ^(*t*)^ was evaluated from simulated SAG sequences. From the simulated distribution of SAG sequences and non-target sequences, the proportions of non-target sequences *p* ^(*n*)^ in the simulated SAG sequences were predicted. The predicted proportions were strongly correlated with the true proportions in both *E. coli* and *M. magneticum* (r = 0.99) (Fig. 6a and d). These results suggest that our method can be used to accurately predict the proportion of contaminant sequences in SAG sequences and cope with various types of target bacteria.

We also predicted the distributions of target sequences *D* ^(*t*)^ by subtracting the distribution of simulated non-target sequences from that of the simulated SAG sequences. When the proportions of the target sequence were more than 20% in the datasets, the predicted distributions were strongly correlated (r > 0.9) with true distributions, although the raw distribution represented much lower correlations (Fig. 6b and e). Therefore, SAG-QC can predict the distribution of target sequences accurately unless the SAG sequences are extremely contaminated.

Confidence scores were estimated based on the predicted contamination rate *p* ^(*n*)^ and distribution of target sequences *D* ^(*t*)^. To examine the performance of the confidence score for predicting target sequences, we generated ROC curves and calculated the AUC. We created ROC curves for confidence scores calculated from the samples simulated with 90% contamination. The AUC was quite high (AUC = 0.986, 0.998) in both *E. coli* and *M. magneticum*. Therefore, these results suggest that our method can be used for the quality control of SAG sequences.

### Performance test with real SAG sequences

We evaluated the performance of SAG-QC using real SAG sequences derived from *E. coli*. We firstly run Kraken and performed taxonomic classification of the SAG sequences. *Pseudomonas*, *Delftia*, *Serratia*, *Stenotrophomonas* and several other taxa were confirmed as contamination from the SAGs of *E. coli* (Additional file 1: Table S1). Those taxa were commonly detected in sequences of no template control, indicating that the contaminating constitients were identical among the SAGs and the no template control.

**Fig. 7:**
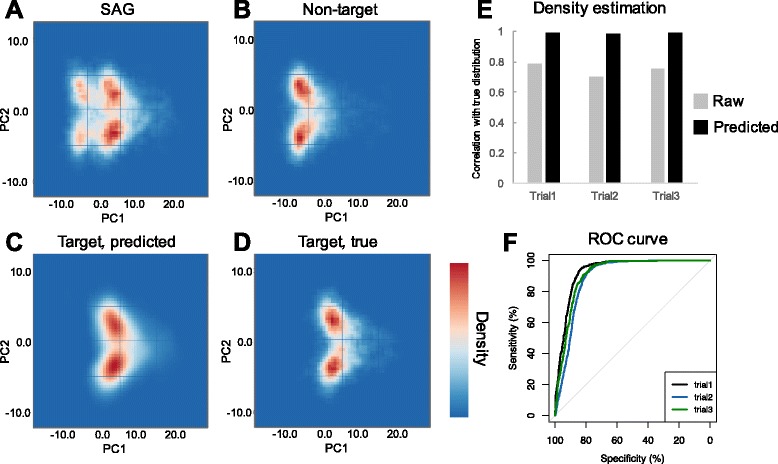
Result of performance test using real SAG data Distribution of (**a**) raw single-cell sequences, (**b**) sequence of negative control, (**c**) target distribution predicted from single-cell sequence, and (**d**) true target distribution calculated based on publicly available genome sequence. **e** Pearson’s correlation between predicted and true target distributions. **f** ROC curve representing accuracy for predicting sequence of the target bacterium

Therefore, we predicted the distribution of target sequences *D* ^(*t*)^ by subtracting the distribution of non-target sequences from that of SAG sequences (Fig. 7a–d). The predicted distributions showed high correlations (r = 0.984–0.990) with the true distribution. Since the correlations were low (r = 0.704–0.788) before subtracting the distribution of non-target sequences, these results suggest that the method enables the accurate prediction of the distribution of target sequences.

Additionally, we calculated confidence scores based on the predicted distribution *D* ^(*t*)^ and the distribution of non-target sequences. ROC curves were generated based on the scores for evaluating their potential to distinguish target sequences from non-target sequences (Fig. 7f). The target and non-target sequences were determined by alignment to the genome sequence of *E. coli.* The target sequences denote sequences aligned to the genome. We observed that confidence scores correctly worked as an indicator (AUC = 0.913). The performance was inferior to the results observed in the test using simulated datasets. This may reflect that the real SAG sequences included artifact sequences produced through MDA. However, our method still showed good performance for extracting target sequences from SAGs without any existing information.

We extracted contigs with confidence scores higher than 70 and evaluated sensitivity and specificity. The sensitivities, proportions of target sequence retained after the selection, were 77.4 – 89.9%. The specificities, proportion of non-target sequences discarded by the selection, were 84.1 – 86.1% (Additional file 2: Table S2). Thereafter, we run ProDeGe on the datasets with a mode not dependent on similarity search and estimated its sensitivity and specificity. Although ProDeGe demonstrated high specificities (96.9 – 98.3%), its sensitivity was much lower (7.3 – 8.7%) than that of our method (Additional file 2: Table S2). We believe this result also promises performance of our method when the target belongs to unexplored taxa and similarity-search-based method is inapplicable.

### Limitation of the method

We used Kraken and confirmed that non-target sequences were derived from various bacterial species (Additional file 1: Table S1). The species were distinct from *E. coli* in order-level (e.g. *Pseudomonas*), class-level (e.g. *Delftia*) and genus-level (e.g. *Serratia*) respectively. Confidence scores were calculated for the non-target sequences. We found that the averages of confidence scores in non-target sequences derived from different class and order were 16.3 and 14.8. They were remarkably low compared to target sequences since its average score was 80.6 (Additional file 3: Figure S1). On the other hand, score of non-target sequences originated from different genera were relatively high. The average and third quantile of the scores were 54.4 and 84.5. Those results indicate the limitation of our approach to distinguish sequences of closely related species. However, the performance would be enough to discriminate non-target sequences derived from different order and class.

In this study, we performed quality control of SAG sequences by using sequences of no template control. It was feasible because contaminating constituents were almost identical between SAGs and no template control (Additional file 1: Table S1). The performance of our tool will be influenced when the contaminating constituents are different between them. The contaminating constituents are possible to be different if no template control was processed independently. Therefore, we strongly recommend users to acquire no template controls in parallel with SAGs under the same experimental conditions (e.g. performing experiments with same reagents in same time and same place) as possible.

## Conclusions

We presented SAG-QC, a computational tool for the quality control of bacterial SAG sequences. SAG-QC possesses functions for both similarities search-based and binning-based quality control methods. In binning-based quality control, SAG-QC utilizes no template control sequences to assign a confidence score to SAG sequences. The confidence score indicates whether the sequence is derived from the target bacterium and can be used as a threshold for extracting sequences during binning. Based on the results of the test using both simulated and real SAG sequences, we demonstrated that the score can be used to distinguish target sequences from SAG sequences. Unlike the conventional metagenomic binning methods [28], our approach can determine bins of target sequences without any existing information. Therefore, the method is available even when a target bacterium belongs to a minor taxonomic group that has not well-studied.

## Availability and requirements

**Project name:** SAG-QC

**Project home page:** https://sourceforge.net/projects/sag-qc/

**Operating systems:** Mac OS X

**Programming language: ** Python

**Licence:** None

## Additional files

Additional file 1: Table S1. Sensitivity and specificity of our approach to discriminate non-target sequences. (XLSX 34 kb)﻿
Additional file 2: Table S2. List of taxa confirmed from contigs of E. coli SAGs and no template control. (XLSX 60 kb)
Additional file 3: Figure S1. Confidence scores for the non-target sequence of different genus, order and class. Box plot representing confidence scores of the non-target sequences derived from *E. coli*, taxa different from *E. coli* in genus-level, order-level and class-level. Lower and upper hinges correspond to the first and third quantiles. (PDF 17 kb)

## Abbreviations

AUC: Areas under curves
MDA: Multiple displacement amplification
ROC: Receiver operating characteristic
SAG: Single-cell amplified genome
WGA: Whole genome amplification
