# Supplementary figures and images for: SAG-QC: quality control of single amplified genome information by subtracting non-target sequences based on sequence compositions
Source: BMC Bioinformatics. 2017 Mar 4;18:152. doi: 10.1186/s12859-017-1572-5 (PMC5336615; doi:10.1186/s12859-017-1572-5)

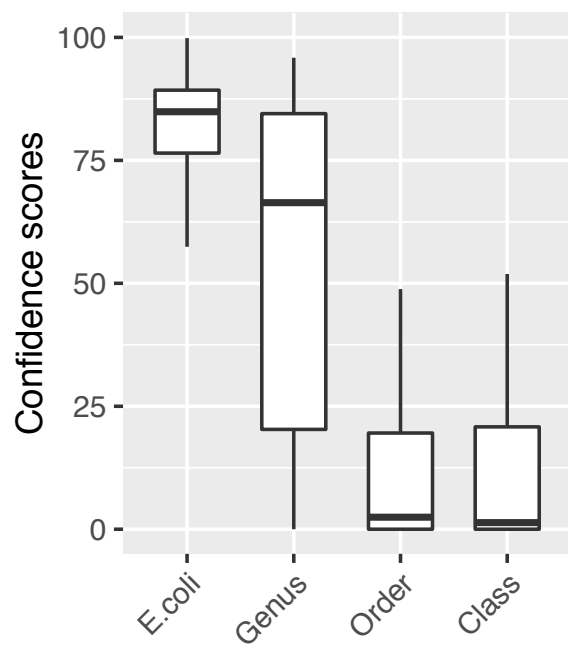

Supplement: Additional file 3: Figure S1. — Confidence scores for the non-target sequence of different genus, order and class. Box plot representing confidence scores of the non-target sequences derived from E. coli, taxa different from E. coli in genus-level, order-level and class-level. Lower and upper hinges correspond to the first and third quantiles. (PDF 17 kb) [file 12859_2017_1572_MOESM3_ESM.pdf]
